# Vaccination

**Published:** 1869-09-15

**Authors:** 


### EDITORIAL.

### *Vaccination.*

In view of the general attention which the subject of vaccination, and especially animal vaccination, is now attracting on both sides of the Atlantic, and, also, of the numerous reports of cases of spurious vaccination, which have recently been published, it seems not inappropriate to consider the subject somewhat, in the abstract, divested of its practical relations, which being objectively the most prominent and apparent have, by attracting the attention and absorbing the thought of observers, obscured the true question at issue, viz.:

Can vaccination be spurious?

The discovery of vaccine by Jenner was an induction from observed phenomena whose identity was an essential prerequisite thereto; had the so-called spurious forms of vaccination existed at that time, with their multiform manifestations, the splendid generalization of Jenner would have been simply impossible. Since the date of its discovery the disease has been studied, in all its phases, so extensively and so minutely that it has become one of the best known and most familiar of the morbid conditions to which humanity is subject, and there is no other whose type is more clearly defined and more accurately described. In the organic world, especially, there is no type absolutely invariable; none in which variations non-essential to its typical integrity may not occur; but these variations must be non-essential, must not involve specific characteristics; if these be modified the specific identity is lost, and the phenomena must be rejected from the typical category.

That which does not present the characteristics of a species does not belong to that species, but must be included in some other. The distinctive features of vaccine having been so clearly defined, and so uniformly recognized, it would seem strange that at this late day in the history of the disease, any doubt whatever could rest upon its identity. That such doubt exists the numerous cases of so-called spurious vaccination abundantly testify. If the evidence upon which the spurious character of these cases rests be examined it will be found to consist of variations from the type in the premonitory symptoms of inoculation, in the appearance of the pustule (local manifestation), or in the consecutive constitutional modifications, or in all of these, and these variations are *specific*. If, moreover, the sources from which the virus has been drawn be investigated they will be found to manifest corresponding variations, will continue to appear in all cases through which the virus may, subsequently, be transmitted. It will happen, frequently, that these variations will present sufficient uniformity of feature to permit their reduction to some well-known category, while, at times, their diversity is so great as to render this difficult or impossible, the evidence being rather negative than positive. A comparison of any of these divergent cases with a typical case of vaccination will always afford negative evidence of a specific character abundantly sufficient to exclude it entirely from that category, and this would appear to be all that was necessary for the pathologist, especially when such comparison may be so readily made with a standard so well defined as typical vaccinia; so that the premonitory symptoms of inoculation, the local disease, and the consequent constitutional changes being carefully determined, there should remain no room for error, no foundation for "spurious vaccination."

In this regard all cases must be classified under the heads vaccinia and non-vaccinia, not "spurious vaccinia," which is, at best, but a facetious epithet for a negation, and should, together with all its congeners, be expunged from scientific nomenclature. By determining accurately, and classifying consistently, that which is, and ignoring that which is not, the whole subject will be enlightened by the removal of the mass of rubbish which now obstructs it.

